# Neurosarcoidosis resembling multiple meningiomas: A misleading presentation of the disease and diagnostic challenge

**DOI:** 10.1177/20584601211036550

**Published:** 2021-07-30

**Authors:** Marta D Switlyk, Pitt Niehusmann, Mette Sprauten, Henriette Magelssen, Mads Aarhus, Finn Ø Rasmussen, Kjetil Knutstad, Petter Brandal

**Affiliations:** 1 Department of Radiology, The Norwegian Radium Hospital, Oslo University Hospital, Oslo, Norway; 2 Section of Neuropathology, Department of Pathology, Oslo University Hospital, Oslo, Norway; 3 Institute of Clinical Medicine (KlinMED), Faculty of Medicine, University of Oslo, Oslo, Norway; 4 Department of Oncology, The Norwegian Radium Hospital, Oslo University Hospital, Oslo, Norway; 5 Department of Neurosurgery, Ullevål Hospital, Oslo University Hospital, Oslo, Norway; 6 Department of Neurology, Ullevål Hospital, Oslo University Hospital, Oslo, Norway

**Keywords:** Neurosarcoidosis, meningioma, dural involvement, magnetic resonance imaging

## Abstract

Sarcoidosis is characterized by the presence of noncaseating granulomatous inflammation in the affected organs. Neurosarcoidosis denotes the involvement of the nervous system and can be either isolated or coexisting with extraneural systemic inflammation. The diagnosis of isolated neurosarcoidosis may be challenging due to unspecific symptoms and similar appearances with other disease processes. This report presents an uncommon case of intracranial sarcoidosis mimicking multiple meningiomas. Familiarity with the spectrum of magnetic resonance imaging findings in neurosarcoidosis is crucial to prevent interpretive errors which may in turn lead to an inappropriate diagnosis and treatment.

## Introduction

Sarcoidosis is characterized by the development of noncaseating granulomatous inflammation in the affected organs.^1,2^ The etiology is unknown; however, the disorder is believed to represent a genetically primed abnormal immune response to antigenic exposure.^3^ Neurosarcoidosis refers to the involvement of the central and/or peripheral nervous system. Extraneural manifestations of the disease are common, whereas isolated neurosarcoidosis is relatively rare, affecting approximately 10%–19% of patients.^1,2^ In this report, we describe an uncommon case of neurosarcoidosis resembling multiple meningiomas. Isolated neurosarcoidosis with focal, dural involvement that mimics an intracranial mass lesion is unusual and can frequently cause interpretive errors in imaging studies.

## Case history

The patient was a 75-year-old woman who underwent gamma knife treatment for a lesion in the right cerebellopontine angle and internal acoustic canal in 2014. The diagnosis was not histopathologically confirmed; however, the lesion was radiologically recognized as a meningioma or vestibular schwannoma. Subsequently, she was regularly followed up at a local hospital, including repeated magnetic resonance imaging (MRI) of the head. During this diagnostic follow-up, multiple dural tumors were diagnosed on MRI, and meningioma was suspected. The lesions were slow-growing and essentially stable in size over the last 12 months.

The patient was referred to our oncology cancer clinic for further management of multiple intracranial lesions, radiologically diagnosed as meningiomas. Due to the development of multiple meningeal tumors, the possibility of underlying neurofibromatosis type 2 (NF2) was evaluated. She had a longstanding history of fatigue, right-sided facial numbness, unsteadiness, and chronic headaches. Despite slight worsening, the patient’s symptoms were not pronounced. Neurological examination revealed right-sided reduced hearing, slight ataxia, and some unsteadiness but was otherwise normal. Some of the symptoms could also be associated with sequelae after gamma knife treatment. The patient was evaluated for radiotherapy, and surgical treatment was not considered because of the multiplicity of the lesions.

**Figure 1.**
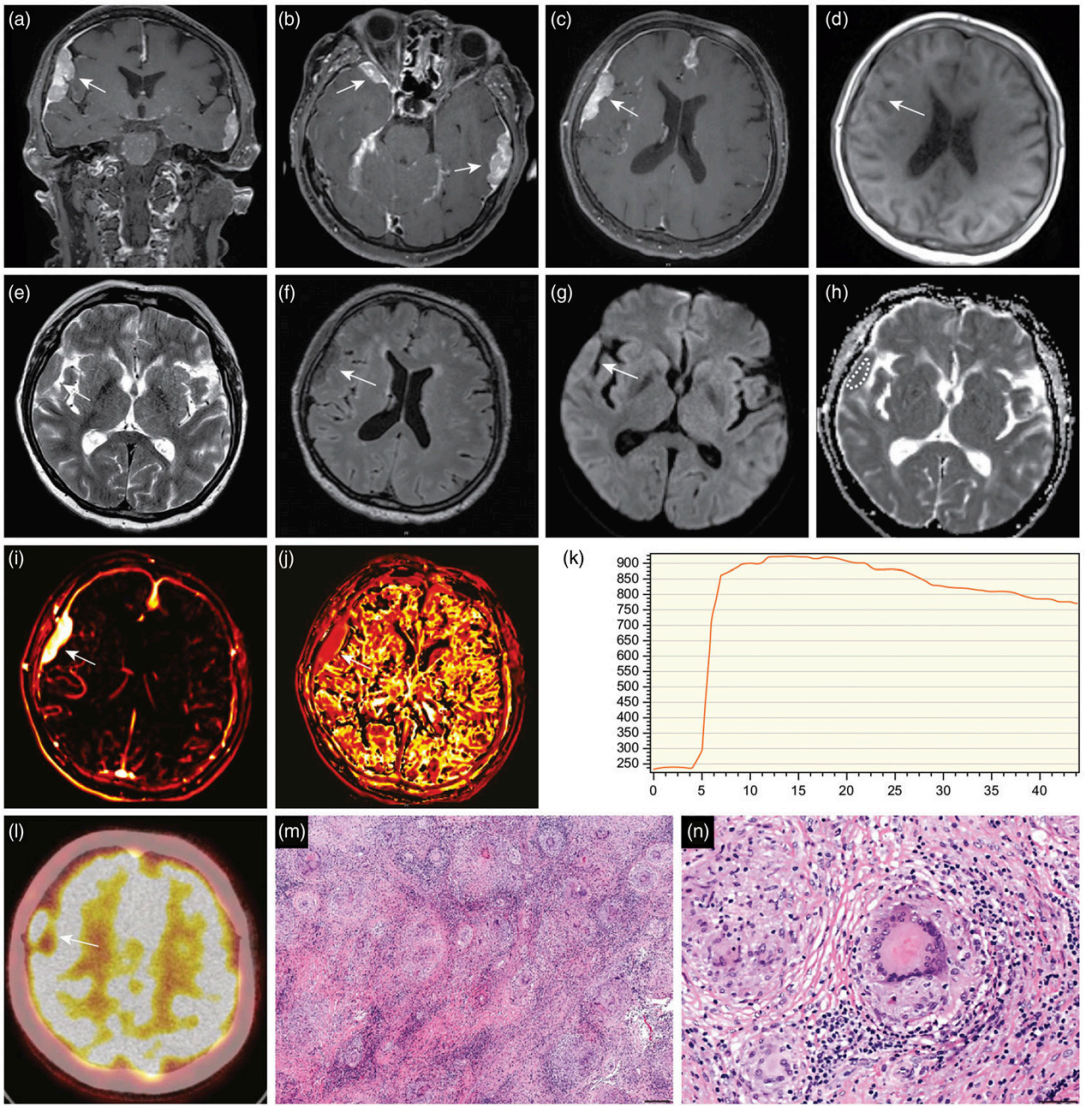
Multiparametric magnetic resonance imaging (MRI) of the head (A)–(K), fused ^18^F-fluorodeoxyglucose positron emission tomography/computed tomography (^18^F-FDG-PET/CT) (l) and biopsy (M), (N) performed in a patient with neurosarcoidosis. Coronal and axial T1-weighted sequences after gadolinium administration show homogenously enhancing, extraaxial lesions (A–C, white arrows). The dural lesions have a solid appearance and low signal on the axial precontrast T1-weighted sequence (D, white arrow), intermediate signal on the axial T2-weighted sequence (C, white arrow), and intermediate/low signal on the fluid attenuated inversion recovery (F, white arrow). Axial diffusion-weighted imaging (b = 1000 s/mm^2^) (G, white arrow) shows a high signal intensity in lesions with a corresponding low apparent diffusion coefficient value (0.8 × 10^−3^mm^2^/s) (H). Dynamic contrast-enhanced MRI (DCE-MRI) derived the transfer constant (*K*^*trans*^) (I, white arrow) and the rate constant (*k*_*ep*_) (J, white arrow) color maps and curve (K) show rapid, initial permeability with some washout. The findings on morphological and functional sequences are undistinguishable from those of a solid tumor. Axial ^18^F-FDG-PET/CT image reveals high tracer uptake in the meningeal lesions (L, white arrow). Histological analysis shows multiple nonnecrotizing, noncaseating granuloma (hematoxylin–eosin stain, scale bar = 200 µm) (M). Higher magnification reveals epithelioid histiocytes, surrounding nonneoplastic lymphocytes and prominent Langhans giant cells with several nuclei arranged in the cell periphery (hematoxylin–eosin stain, scale bar = 50 µm) (N). The biopsied lesion is shown in panel (C)–(J) (white arrows).

**Figure 2.**
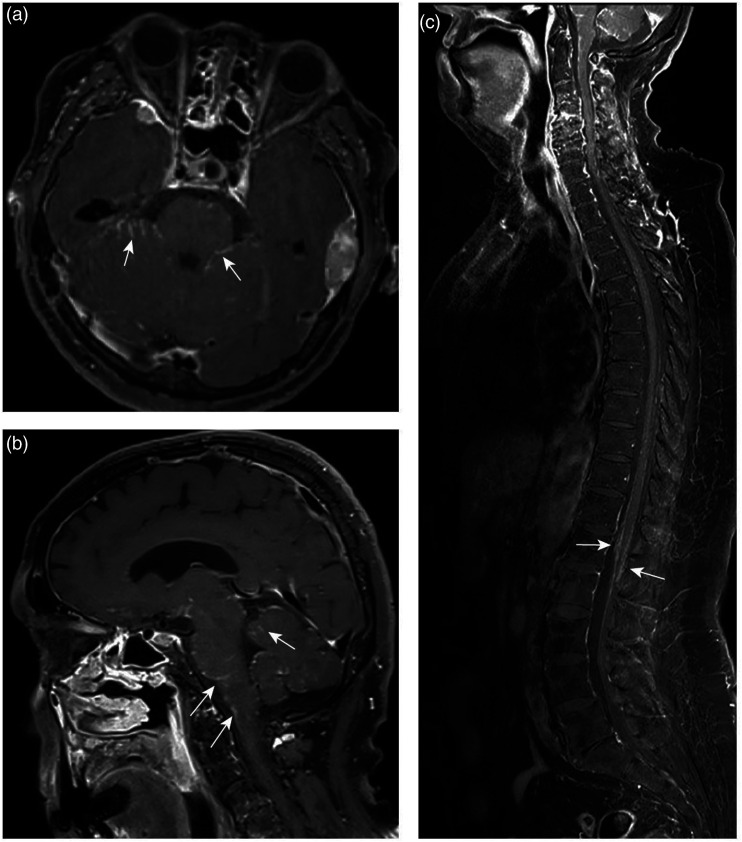
Axial and sagittal, postgadolinium T1-weighted MRI of the head reveals subtle, nodular thickening and enhancement of the leptomeninges, most pronounced infratentorial (A, B, white arrows). Sagittal, postgadolinium *water-only* T1 Dixon MRI of the whole spine shows leptomeningeal enhancement along the spinal cord and cauda equina (C, white arrows).

A new multiparametric MRI of the head was performed, which revealed multiple dural masses along the skull base and convexities (Figure 1). There was no perifocal edema or mass effect. The lesions had a solid appearance, homogenous contrast enhancement, and an intermediate T2 signal. The functional sequences presented moderately low diffusion and rapid initial permeability on dynamic contrast-enhanced MRI (DCE-MRI) perfusion. The dural tail sign was observed adjacent to most of the lesions. In addition to these findings, MRI revealed subtle, leptomeningeal enhancement supra- and infratentorial with symmetrical, deep, and superficial distributions (Figure 2). The dural masses had a solid tumor appearance, and the findings on morphological and functional MRI sequences closely resembled those of meningiomas. However, the coexisting leptomeningeal enhancement was atypical and of uncertain etiology. Possible differential diagnoses included inflammatory, granulomatous meningitis, and carcinomatous meningitis with leptomeningeal spread from an unknown primary cancer. Further radiological workup was performed, including contrast-enhanced MRI of the spinal canal and whole-body ^18^F-fluorodeoxyglucose positron emission tomography/computed tomography (^18^F-FDG-PET/CT). Spinal MRI showed diffuse, leptomeningeal enhancement along the spinal cord and cauda equina (Figure 2). Whole-body ^18^F-FDG-PET/CT revealed high tracer uptake in known meningeal lesions, along with high uptake in small, irregular nodule in the left breast and enlarged left axillary lymph node.

**Figure 3.**
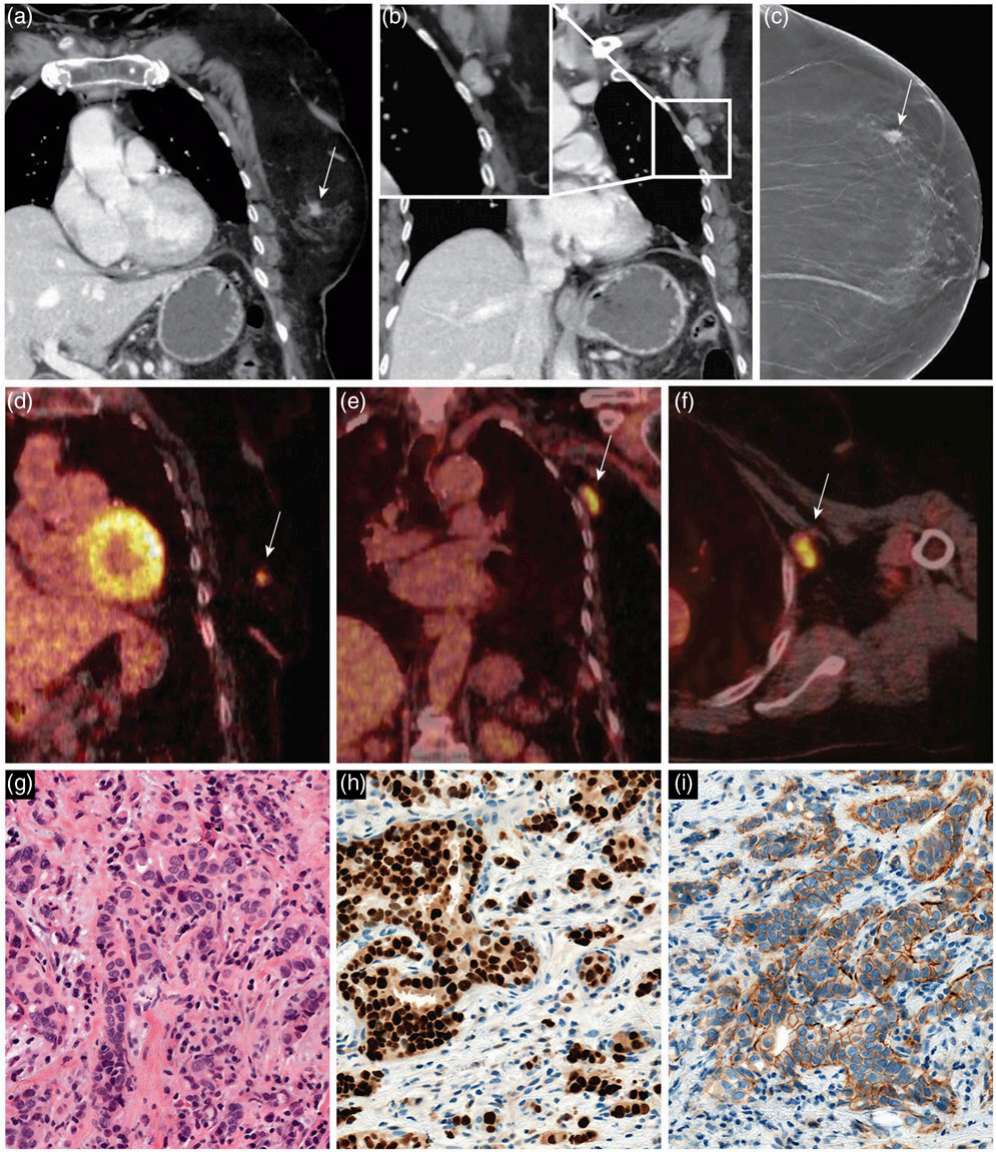
Coronal computed tomography (CT) and fused ^18^F-fluorodeoxyglucose positron emission tomography/computed tomography (^18^F-FDG-PET/CT) shows high tracer uptake in a small, irregular mass in the left breast and an enlarged left axillary lymph node (A, B, D–F, white arrows). The supplementary mammogram shows a spiculated mass in the left breast consistent with malignancy (C, white arrow). The biopsy of the tumor in the left breast and axillary lymph node confirmed infiltrating breast carcinoma with nodal metastasis. Tumor was estrogen receptor (ER)-positive and human epidermal growth factor receptor 2 (HER-2)-negative (hematoxylin-eosin stain, scale bar = 50 µm) (G), (ER, scale bar = 50 µm) (H), (HER-2, scale bar = 50 µm) (I).

The supplementary mammogram revealed a spiculated mass in the left breast consistent with malignancy. Biopsy of the breast tumor and axillary lymph node confirmed the presence of infiltrating carcinoma with nodal metastasis (Figure 3). The tumor was histologically assessed as grade 2 carcinoma of no special type (NST) with the presence of estrogen (ER 100%) and progesterone receptors (PgR 100%) and was human epidermal growth factor receptor 2 (HER-2) negative.

Laboratory tests revealed an elevated erythrocyte sedimentation rate (ESR) of 79 mm (normal range 1–17 mm) and a slightly elevated C-reactive protein (CRP) level of 9.6 mg/L (normal range <4 mg/L); all other results were within normal limits. Cerebrospinal fluid (CSF) analysis revealed pleocytosis of white blood cells (WBC) 50 × 10 × 10^6^/L (normal range 0–4 × 10 × 10^6^/L) and elevated protein level of 3.53 g/L (normal range 0–0.45 g/L), while CSF cytology and culture results were negative.

Due to the sustained diagnostic dilemma of the etiology of the intracranial lesions, a biopsy of the right frontal tumor was obtained. Somewhat surprisingly, the biopsy showed epithelioid histiocytes, abundant multinucleated giant cells, and surrounding nonneoplastic lymphocytes and plasma cells. Ziehl–Neelsen staining revealed no evidence of acid-fast bacilli. The findings were consistent with nonnecrotizing, noncaseating granulomatous inflammation and were compatible with neurosarcoidosis (Figure 1).

**Figure 4.**
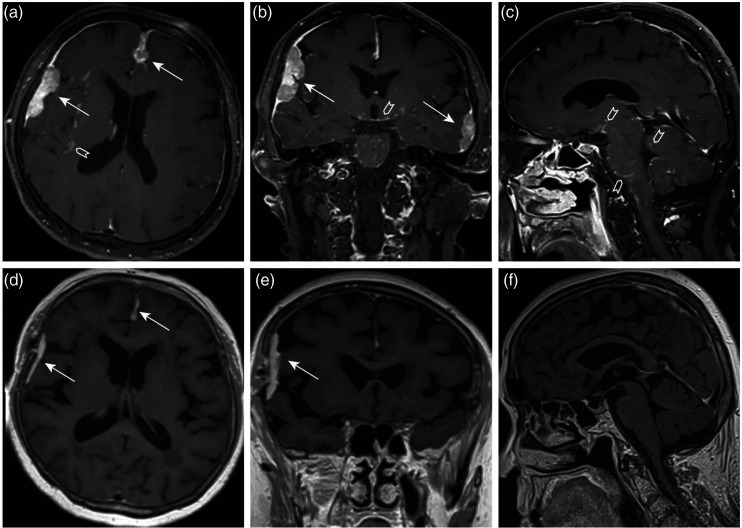
The pretreatment magnetic resonance (MR) images are presented in the upper row (A)–(C). Axial, coronal, and sagittal postgadolinium T1-weighted sequences show multiple dural lesions (white arrows) and subtle leptomeningeal contrast enhancement (white arrowheads) consistent with neurosarcoidosis. The follow-up MR images performed 2 months after the initiation of corticosteroid therapy show a good treatment response in dural lesions (D, E, white arrows) and complete resolution of the leptomeningeal enhancement (D)–(F).

Since symptoms related to neurosarcoidosis were not profound, anti-neoplastic treatment was prioritized. The patient underwent uncomplicated breast-conserving surgery with left axillary lymph node dissection for incidentally detected breast cancer. Furthermore, she was scheduled for the initiation of corticosteroid therapy for neurosarcoidosis. The follow-up MRI of the head performed 2 months after corticosteroids initiation showed a good treatment response in dural lesions and complete resolution of the leptomeningeal contrast enhancement (Figure 4).

## Discussion

Sarcoidosis is characterized by the presence of a granulomatous inflammatory reaction in the affected organs, including the central nervous system (CNS).^4,5^ Neurosarcoidosis can coexist with extraneural manifestations or, less commonly, can be isolated. Clinical symptoms are often nonspecific and depend on the location of granuloma involvement in the CNS.^6^ Gadolinium-enhanced MRI is the gold standard for neurosarcoidosis imaging. Intense physiological uptake in the brain limits the evaluation of neurosarcoidosis using ^18^F-FDG-PET/CT; however, this modality can be used for staging, detection of extraneural lesions, and determination of the optimal biopsy site.^7,8^ The disease can involve practically any part of the nervous system and its coverings, including the brain and spinal parenchyma, nerve roots, leptomeninges, dura mater, and the surrounding bony structures.^6^ The wide spectrum of imaging findings in neurosarcoidosis is well documented, and the entity can mimic numerous inflammatory, infectious, autoimmune, and neoplastic processes.^9,10^ The list of differential diagnoses to be considered for dural sarcoidosis includes meningioma, solitary fibrous tumor, hemangiopericytoma, dural metastases, lymphoma, leukemia, plasmocytoma, Rosai–Dorfman disease, Wegener’s granulomatosis, idiopathic hypertrophic cranial pachymeningitis, and granulomatous infection.^11,12^ Meningioma is the principal differential diagnosis for slow-growing dural lesions, and two familial syndromes linked to multiple meningiomas are NF2 and familial meningiomatosis.^13^ A summary of published cases of intracranial sarcoidosis mimicking meningioma is presented in Table 1.

**Table 1.** Intracranial sarcoidosis mimicking meningioma: Summary of relevant cases in the literature.

First author	Case summary				
	Age/gender	Symptoms	Imaging modality	Number of lesions	Location of lesions
Jackson et al.^16^	44/F	Headaches, decreased memory, diminished energy, personality changes, and decreased coordination	MRI	Multiple	Parafalcine and bilateral frontoparietal mass
Rodriguez et al.^14^	45/F	Hearing loss, left ear pain, and sensory loss on the left side of face	CT and MRI	Solitary	Middle cranial fossa, cavernous sinus, Meckel cave, cerebellopontine angle, and internal acoustic canal
Lipper et al.^17^	42/F	Nausea, gastric fullness, dizziness, acute decrease in hearing in right ear, and tinnitus	CT and MRI	Solitary	Petrous apex and petroclinoid ligament, clivus, and internal acoustic canal
Weil et al.^18^	46/F	Mild right arm and leg weakness, word-finding difficulties, and left–right discrimination errors	MRI	Solitary	Parietal region
Osenbach et al.^19^	34/M	Right temporal and retro-orbital headaches and seizures	CT	Solitary	Sphenoid ridge and contiguous convexity
Wilson et al.^15^	30/F 30/M 37/F	Generalized seizures, headache, dizziness, and gait disturbance Headache, diplopia, and decreased vision Recent onset of decreased vision	CT and MRI	Multiple Solitary Solitary	Dural lesions along convexities Cerebellopontine angle, sella, parasellar region Middle cranial fossa, cavernous sinus, anterior clinoid process, optic nerve
Sandhu et al.^20^	48/F	Syncopal episode and blurred vision	CT and MRI	Solitary	Temporal convexity mass
Strickland-Marmol et al.^21^	52/F	Left-sided headache and shooting pain on left side of face	CT and MRI	Solitary	Temporal region and cavernous sinus
Ranoux et al.^22^	32/F	Left-sided retro-orbital headache and paresthesia of the left side of face	CT and MRI	Solitary	Cavernous sinus
Tan et al.^23^	62/F	Hearing loss, tinnitus, dizziness, and ataxia	MRI	Multiple	Posterior cranial fossa and tentorium
Wang et al.^24^	27/M	Generalized seizures	MRI	Multiple	Sphenoid wing and temporal and occipitoparietal regions
Nowak et al.^25^	24/M	Recurrent seizures	MRI	Multiple	Frontal and temporal regions

Abbreviations: CT, computed tomography; F, female; M, male; MRI, magnetic resonance imaging.

Potential MRI findings in neurosarcoidosis include nonenhancing or enhancing intraparenchymal lesions, leptomeningeal involvement, hypothalamus and pituitary involvement, cranial nerve involvement, dural involvement, and vasculitis-like lesions.^6^ Leptomeningeal involvement (sarcoid meningitis) is possibly the most typical manifestation of neurosarcoidosis, seen in approximately 40% of cases.^6^ Leptomeningeal sarcoidosis commonly manifests as diffuse or nodular thickening and enhancement of the leptomeninges on MRI.^1^ The finding is nonspecific and imaging studies can hardly distinguish sarcoidosis from carcinomatous, lymphomatous, or infectious meningitis. Dural involvement is less common, and the presentation of single or multiple, tumor-like, extraaxial masses is fairly unusual, particularly without coexisting extraneural manifestations.^14^ In our patient, combined leptomeningeal and dural involvement was present, which is also uncommon. The low rate of occurrence of dural and leptomeningeal disease in the same location can be explained by the presence of arachnoid barrier cells that slow down or prevent the spread of disease through the arachnoid membrane.^6,11^ Meningeal lesions in dural sarcoidosis typically have very low T2 signals on MRI.^15^ This hypointensity has been reported previously and is probably related to fibrocollagenous buildup.^11,15^ Occasionally, as in our case, T2 hyper or isointensity resembling a solid tumor is noted, probably due to inflammation rather than fibrocollagenous/gliotic tissue.^10^ Functional MRI findings of sarcoid granuloma in our patient were undistinguishable from those of solid tumors with moderately low diffusion and rapid initial permeability with some washout on DCE-MRI perfusion. This highlights the importance of tissue sampling and histological confirmation in sarcoidosis, in addition to clinical and radiological features.

Sarcoidosis is known to be a great mimicker, and its diagnosis can frequently be delayed due to nonspecific clinical symptoms and interpretive errors in reading imaging studies. Our case demonstrates an unusual and misleading presentation of isolated neurosarcoidosis with combined leptomeningeal and dural involvement. The dural lesions were relatively stable over time and could not be distinguished from meningiomas on morphological and functional MRI sequences. Evaluation of leptomeningeal involvement was additionally challenging because of incidentally detected metastatic breast carcinoma. However, the negative CSF cytology and good treatment response after the initiation of corticosteroid therapy were strongly suggestive of neurosarcoidosis.

In conclusion, an accurate diagnosis of neurosarcoidosis strongly depends on multidisciplinary collaboration between clinicians, neuroradiologists, and neuropathologists. Although uncommon, neurosarcoidosis should be kept in mind as an important differential diagnosis for ambiguous meningeal lesions, and the threshold of tissue sampling should be low. Moreover, familiarity with the spectrum of imaging findings is crucial for appropriate diagnosis and treatment.
